# It Suddenly Occurred: Extensive Subcutaneous Emphysema after Bipolar Transurethral Resection of Prostate

**DOI:** 10.1155/2015/134651

**Published:** 2015-09-28

**Authors:** Murat Bagcioglu, Mert Ali Karadag, Ramazan Kocaaslan, Cafer Mutlu Sarikas, Mustafa Gok, Omer Erkam Arslan

**Affiliations:** ^1^Urology Department, Faculty of Medicine, Kafkas University, 36000 Kars, Turkey; ^2^Anesthesiology and Reanimation Department, Faculty of Medicine, Kafkas University, 36000 Kars, Turkey; ^3^Radiology Department, Faculty of Medicine, Kafkas University, 36000 Kars, Turkey

## Abstract

Subcutaneous emphysema is a very rare and good-natured complication after transurethral resection of prostate (TURP). It has been reported as colon perforation, diverticulitis, and bladder perforation associated complication previously. We report the first case of a wide subcutaneous emphysema due to microperforations of prostatic capsule, without a bladder perforation after TURP. Any sign of clinic situation should lead to ceasing of the procedure immediately; otherwise, it can cause a life-threatening problem of abdominal compartment syndrome.

## 1. Introduction

Transurethral resection of prostate (TURP) is the gold standard in surgical management of benign prostatic hyperplasia (BPH), and it is with decreased morbidity and mortality rates in the last decades, but it is still not without complications [1]. TURP involves several types of complications such as hematuria, urinary infection, extraperitoneal and intraperitoneal extravasation of irrigation fluids, transurethral resection syndrome, bladder perforation, injury of external sphincter or ureteral orifices, and even mortality [2]. Herein, we present a case of subcutaneous emphysema on abdominal wall, chest wall, and face due to microperforations of the prostatic capsule with access of air to the retroperitoneal space. To the best of our knowledge, this rare complication is the first report of such a wide subcutaneous emphysema without a bladder perforation after TURP.

## 2. Case Presentation

**Figure 1:**
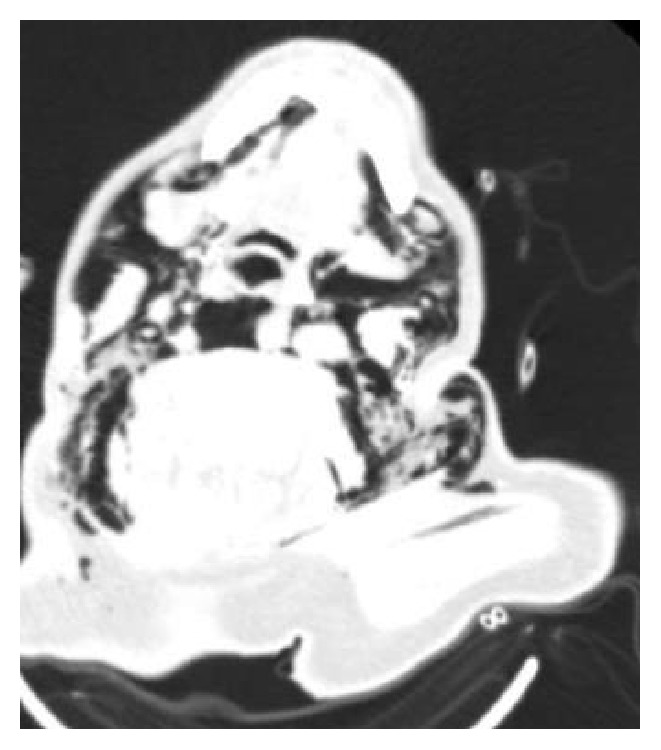
Extensive air density in all spaces of the suprahyoid neck region.

**Figure 2:**
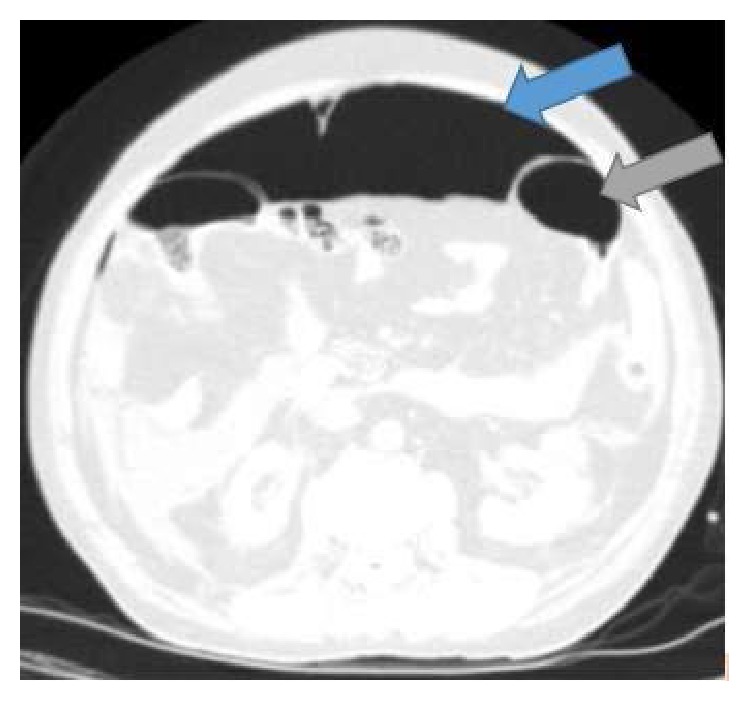
Massive pneumoperitoneum (with blue arrow) and normal intraluminal intestinal air density (with grey arrow).

A 70-year-old man presented with lower urinary tract symptoms and gross hematuria. He had no specific medical history. Pertinent laboratory tests included a hematocrit of 24.3%, hemoglobin level of 8.2 g/dL, and creatinine of 0.9 mg/dL. Abdominal ultrasound revealed that the prostate was approximately 100 g, and there was 58 × 43 mm thrombus in the bladder without any significant hydroureteronephrosis. The patient was hospitalized and a 22 Fr Foley catheter was inserted. The bladder was irrigated to remove all the clots, which could cause catheter blockage, and the continuous bladder irrigation was used. The patient was scheduled to undergo cystoscopy and TURP. Preoperative chest X-ray findings were normal and electrocardiography showed a normal sinus rhythm. At the operating room, the blood pressure was 115/85 mm Hg, heart rate was 92 bpm, and oxygen saturation was 98%. The surgical procedure started under spinal anesthesia with a 20 Fr cystoscope (Storz, Germany). After bladder irrigation with saline solution, approximately 400 mL of blood clot was removed by an evacuator. A 24 Fr resectoscope using the Plasma Kinetic Tissue Management System (Gyrus Medical, Bucks, UK) with a bipolar electrosurgical device was inserted after evacuating. The cutting power was adjusted as 200 W, and a TUR loop of 80/20 platinum/iridium was used during the procedure. 0.9% NaCl saline solution was used as irrigation fluid, typically only lifted 50 cm above the patient's level. Approximately 110 minutes after the beginning of the surgery, 80 grams of prostatic adenoma was resected completely, but the patient felt serious discomfort and abdominal and thoracic pain; an oxygen saturation decline to 94% was noticed, and the surgery was ceased immediately. The drapes were removed, and the abdomen was noticed to be distended and tympanic to percussion. A crackling feeling around the chest wall, neck, and face was revealed. An immediate cystoscopic exploration was performed for suspicion of a bladder rupture, but it was excluded. A cystogram showed some contrast material leakage at the site of the prostatic capsule, without any leakage from bladder. An urgent computerized tomography (CT) scan of thorax, abdomen, and pelvis was performed, and it showed retroperitoneal fluid collection and massive air, extensive subcutaneous emphysema on chest wall, neck, and face (Figures 1 and 2).

We decided a conservative treatment, and the patient was transferred to intensive care unit for close monitorization. The patient was hemodynamically stable at follow-up, subcutaneous emphysema disappeared, and abdomen was soft during the 4 days after surgery. The Foley catheter was removed on the fourth day, and the patient was discharged from the hospital on the fifth day after the surgery.

## 3. Discussion

The bipolar technology in TURP was introduced to avoid transurethral resection syndrome and to increase resection time. In the TURP, a prolonged operative time was defined as an independent risk factor for surgery related complications; a 2.4% increased risk of perioperative complications per minute resection was observed [3]. Microperforations of the prostatic capsule in TURP are not very rare during this surgical procedure. Extraperitoneal fluid collection is one of the common complications after TURP, which was classified using the Clavien system [3]. Also, two prior cases with abdominal compartment syndrome and pubic osteomyelitis due to capsular perforation after prostatectomy were reported before [4, 5]. Pneumoretroperitoneum, pneumomediastinum, and subcutaneous emphysema were reported as colon perforation or diverticulitis and bladder perforation associated complication previously [6–8]. In our case, this clinic situation happened due to microperforations of prostatic capsule, without any bladder and colonic injury, and probably the overaggressive irrigation of the bladder preoperatively.

Air can be entered into the bladder via cystoscope insertion, irrigation tube, instrument manipulation, and evacuator usage. Also, the use of diathermy like bipolar energy in bladder can cause collection of gases. The air and gas are entered from the microperforations of prostatic capsule through the retroperitoneal pelvic compartment, great vessels, through behind the crus, aortic hiatus, and caval foramen of the diaphragm, and then into the mediastinum, neck, and face [8]. Another way for air passage from retroperitoneum to subcutaneous tissue is the connected fascial planes, which permits air arising to even face [9].

An opening defect or an abnormal hiatus such as an anatomical variation can be another reason for wide spread of air and gas for this patient.

Subcutaneous emphysema and extraperitoneal collection of irrigation fluid are not a life-threatening problem. It can be managed with conservative treatment such as close monitoring, diuretic usage, pain control, and supplemental oxygen therapy [10]. However, in case of abdominal compartment syndrome due to massive fluid collection, which is characterized by increased intra-abdominal pressure, decreased venous return, and hypovolemic shock [11], percutaneous drainage or laparotomy should be considered to decrease morbidity and mortality.

## 4. Conclusion

While subcutaneous emphysema is a very rare and good-natured situation, to avoid this complication, the operating room team should examine the abdomen, chest wall, neck, and face by inspection and palpation intermittently during prolonged transurethral resection procedures. Any sign of clinic situation should lead to ceasing of the procedure immediately; otherwise, it can be cause for a life-threatening problem of abdominal compartment syndrome.
